# Increasing exposure to global climate change and hopes for the era of climate adaptation: An aquatic perspective

**DOI:** 10.1007/s13280-024-02125-1

**Published:** 2025-01-13

**Authors:** Karsten Rinke, Chenxi Mi, Madeline R. Magee, Cayelan C. Carey

**Affiliations:** 1 Department of Lake Research, Helmholtz Centre for Environmental Research, Brückstraße 3A, 39114 Magdeburg, Germany; 2 College of Water Conservancy, Shenyang Agricultural University, Shenyang, China; 3 Wisconsin Department of Natural Resources, 101 S Webster Street, Madison, WI 53703 USA; 4 Department of Biological Sciences, Virginia Tech, 926 West Campus Drive, Blacksburg, VA 24061 USA; 5 Center for Ecosystem Forecasting, Virginia Tech, 1015 Life Science Circle, Blacksburg, VA 24061 USA

## Introduction

The Anthropocene is characterised by technological innovations, increases in industrial productivity, accelerating globalisation, and a rising computer- and information-based cultural evolution. At the same **time**, humankind is leaving significant footprints on its environment, with pollution approaching or even trespassing planetary boundaries. Since the 1980s, accelerating climate change has been severely affecting biomes and Earth’s systems with increasing intensity.

The evidence for climatic changes on a global scale is overwhelming and thoroughly documented through myriad scientific publications, as well as the comprehensive information delivery of the Intergovernmental Panel on Climate Change (IPCC 2023). Compared to other environmental crises, we are well-informed, with broad scientific evidence available for the ongoing changes in our climate system.

As a result, we are all living in the era of climate change. Societies, economies, and cultural attitudes are undergoing transformation, and we need to adapt to the rapidly changing conditions in our natural, societal, and cultural environments. In that sense, the era of climate change is transitioning into the era of climate adaptation! Scientists are not only asked to provide evidence of climate change and its catastrophic impacts but also to deliver solutions for climate adaptation. It is a bit of a frustrating drama that researchers who were initially engaged in documenting climate change and warning about its consequences, remained mostly unheard by decision-makers and are now asked to deliver solutions for climate change adaptation and mitigation.

**Fig. 1:**
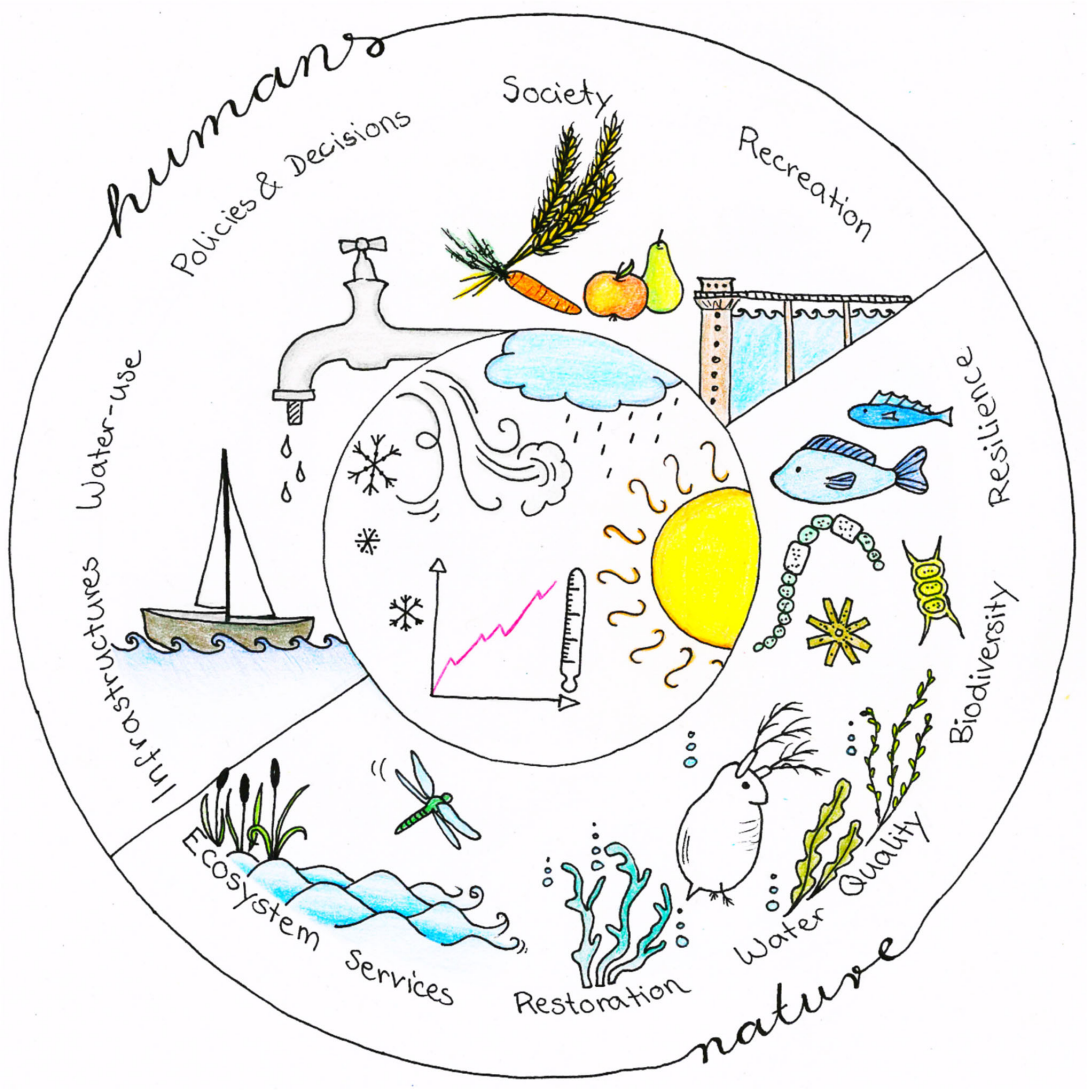
Water for humans and nature. Illustration by Judith Rinke

Aquatic ecosystems, the ecosystem services they deliver, and their use by humans are especially vulnerable to climate change (IPCC 2023). The water sector needs to rethink existing policies and management practices with respect to their validity under changed climatic conditions. Water is required by both humans and ecosystems (Fig. 1) to sustain food production, water supply, and tourism, while also ensuring resilient aquatic ecosystems with rich biodiversity and ecosystem services. Maintaining this balance is extremely challenging, which is made even more difficult under highly uncertain conditions, as our climate future remains unclear. “Climate-proof” freshwater management therefore has to include uncertainties and the special consideration of extreme events, possibly deviating far from historical conditions. It is thus critical that scientists contribute to making water management more climate-proof and to sustaining, protecting, and restoring aquatic ecosystems through the integrated work of several disciplines.

### Footprints of climate change on our inland waters and their impacts on societal welfare

Our focus is on aquatic environments because of their particular sensitivity to climate change. First, they are exposed to increasing water loss by evaporation (Zhao et al. 2019, 2022), and many smaller water bodies undergo transition from permanent to intermittent aquatic environments (Messager et al. 2021). The effects of increasing water stress impact human society, with the most stressed basins being home to 1.5 billion people and delivering about 17% of global food crop production (Huggins et al. 2022). At the same time, climate change also induces increasing flood risks (Blöschl et al. 2019, 2020), requiring humans to adapt to both water shortage and water excess. This is pointing to the importance of storage capacities, such as in the form of reservoirs.

But it is not just about water quantity. Climate-induced effects are deteriorating water quality, degrading aquatic ecosystems, and disrupting ecosystem service provisioning. In large-scale global assessments, current and future water scarcity in many areas is not just a shortage of water but also a result of restricted water resources utilisation due to poor water quality (Vliet et al. 2021; Jones et al. 2024) and limited ecosystem service delivery (Janssen et al. 2021). Typical indicators of climate-induced water quality deterioration include intensifying harmful phytoplankton blooms (Ho et al. 2019; Feng et al. 2024), deoxygenation (Jenny et al. 2016; Jane et al. 2021), and exposure to extreme heatwaves (Woolway et al. 2021a, b). In many cases, the symptoms of climate warming are similar to those of eutrophication (e.g. Moss et al. 2011; Meerhoff et al. 2022), as warming can accelerate nutrient cycling and prolong vegetation periods.

This increase in lake productivity and algal blooms has broad implications for lake management. On the one hand, it implies that lakes exhibit eutrophication-like symptoms, even if no increase in external nutrient loading occurs. This can push lakes into a state of accelerating eutrophication once the decisive tipping point of deep water anoxia is reached and internal loading intensifies (Tammeorg et al. 2020; Lewis et al. 2024). Moreover, any achieved progress in restoration of aquatic environments may become offset by climate-driven eutrophication. Therefore, we may need to refine our restoration targets towards stricter pollution controls and lower nutrient loading thresholds.

The high value of inland waters for recreation, tourism, property values, and sports decreases when water quality declines (Dodds et al. 2009; Weng et al. 2020), e.g. through the occurrence of harmful algal blooms. The costs of drinking water production rise (Pretty et al. 2003) even at the initial stages of eutrophication, because of increases in algal biomass, bacterial load, or release of manganese from sediments. Finally, all water quality deterioration and corresponding management challenges will ultimately incur greater costs and interfere with ecosystem service provisioning (Keeler et al. 2012).

### Climate change calls for adaptation across multiple lines of action

**Fig. 2:**
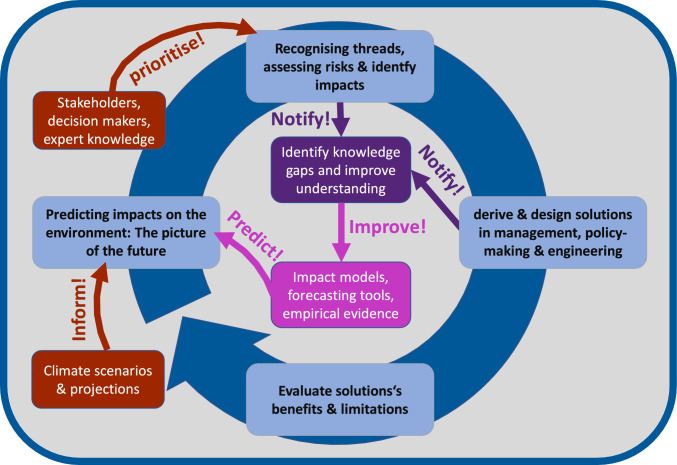
Cycle of climate adaptation research

Willingness to invest in adaptation and counteract further deterioration requires a sound knowledge base that goes beyond pure science and needs to include societal processes, decision-making, and engineered solutions. Climate adaptation therefore demands a whole chain of actions that includes climate projections with fully specified uncertainties, predicting their effects in the environment, identifying solutions, and assessing their capacity as well as stakeholder and societal integration (Fig. 2). Managers of inland waters require concrete, quantitative, and reliable information along with sophisticated tools and process knowledge arising from tight co-development between practitioners and researchers (e.g. Carey et al. 2022). This takes time. Adapting ourselves and our environment to the dramatic changes ahead requires staying power, persistence, courage to experiment, continuous optimisation, and plenty of patience.

Climate adaptation research often includes optimisation steps and ongoing adjustments so that a research cycle comes into play with different components and co-development between research disciplines, as well as involvement of experts, policy-makers, societal groups, and other stakeholders (Fig. 2). An important starting point of this cycle is state-of-the-art climate projections at time scales of years and decades, which together with so-called impact models (e.g. a lake model) can be translated into potential futures of the respective sector or environmental compartment (in this case, a lake). Based on such projected future status of the environment, tailored adaptation strategies can be invented, planned, and tested together with practitioners and stakeholders. The assessment of alternative adaptation strategies is important and may take place based on simulations, laboratory experiments, or field applications to assess their efficiency.

Whenever reliable models are available, an assessment purely based on model scenarios is feasible (e.g. see Mi et al. 2025 in this Special Section; Olsson et al. 2025 in this Special Section). But for climate adaptation efforts characterised by lower levels of predictability (e.g. because they include social systems), assessment cannot be done purely through modelling and requires field work or practical testing in the target environment (e.g. see Tran et al. 2025 in this Special Section). Finally, existing knowledge gaps are usually identified, particularly when concrete solutions are required or even implemented. In the optimal case, these knowledge gaps can guide future research and lead to improved process knowledge (Fig. 2).

## Conclusions

Traditional water management needs an update towards developing and implementing new adaptation strategies that mitigate negative impacts of climate change, make freshwater ecosystems more resilient, and secure human use. A thorough concept of climate-proof water security hence entails water for humans as well as water for the environment with equal priority. Climate adaptation of our inland waters, therefore, requires multi-level activities. It includes proper process knowledge and reliable predictions as scientific basis, robust water management strategies, adjusted infrastructures for new engineering strategies, as well as societal integration and wise decision-making for sustainable socioeconomic welfare. In the ideal case, human water uses for water supply, food production, recreation, and the corresponding infrastructures do not harm biodiversity, self-purification, and ecosystem health (see cover illustration of this issue). Whether this becomes real or remains an unrealised utopia will be one of the key challenges of our century.

In general, climate adaptation should be proactive and not reactive. This implies that scientists can foresee the major risks early enough so that adaptation can be achieved before the damage becomes real. We need to solve a problem before it occurs! Reliable forecasts with specified uncertainty are therefore the decisive prerequisite to successful climate adaptation. If forecasting fails, adaptation does so as well.

This Special Section explores novel strategies for climate adaptation and impact mitigation for freshwater ecosystems, including water resources management, water governance, and new instruments for science-based decision-making (Table 1). Different aspects of climate-proofing for our freshwaters involve, for example, the following research efforts:

- Inventing adaptive and robust engineering strategies
- Providing decision-support and reliable forecasts to managers
- Stabilising ecosystem service provisioning from aquatic environments
- Identifying climate-proof restoration targets for managers
- Adjusting social, economic, and regulative policies towards climate adaptation

**Table 1:**
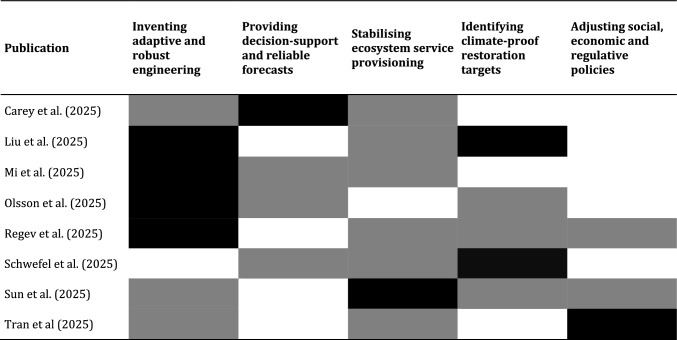
Aspects of multi-level research of the contributed publications in this special section; black: primary focus; grey: secondary focus

The contributions to this Special Section of *Ambio* all embrace at least three of these aspects, although each publication has its own focus. These articles exemplify how climate adaptation research benefits from multi-level approaches that include contributions to process knowledge, management, modelling, restoration, and other fields relevant to sustaining aquatic ecosystems.
